# Impact of thermodynamics and kinetics on the carbon capture performance of the amine-based CO_2_ capture system

**DOI:** 10.1007/s11356-024-33792-y

**Published:** 2024-05-31

**Authors:** Turkan Kopac, Yaşar Demirel

**Affiliations:** 1 Department of Chemistry, Zonguldak Bülent Ecevit University, 67100 Zonguldak, Türkiye; 2 Department of Chemical and Biomolecular Engineering, University of NE—Lincoln, Lincoln, NE USA

**Keywords:** Amines, Aspen plus modeling, Carbon capture, Carbon dioxide, CO_2_ capture, Chemical absorption, Decarbonization

## Abstract

Solvent-based CO_2_ capture is a commonly employed post-combustion technique in processes involving absorber-stripper columns. This study focused on computer simulations with equilibrium- and rate-based modeling of CO_2_ capture using the amine solvents 2-amino-2-methyl-1-propanol (AMP), diethanolamine (DEA), and methyl diethanolamine (MDEA) and thermodynamic methods involving electrolyte NRTL models. The objective of this study was to understand the impacts of rate-based modeling, the type of amine, and thermodynamic methods on carbon capture. Within this study, the amine-based CO_2_ capture process from coal-power plant flue gas was studied using Aspen Plus modeling. Simulations were also conducted to determine the impact of thermodynamics and kinetics on the CO_2_ capture performance of the system. The results were analyzed on the basis of captured CO_2_ according to the solvents and models. The equilibrium approach was mostly invalid because of the oversimplified ideal stage assumptions through the column. The lowest carbon capture capacity was obtained with MDEA, while DEA yielded the best results. A sensitivity analysis with rate-based modeling showed the significant impact of the inlet CO_2_ composition. The amine-based CO_2_ capture process simulation included solution chemistry, electrolyte thermodynamics, rigorous transport property modeling, reaction kinetics, and rate-based multistage simulation, which could be applicable to different solvent systems.

## Introduction

### Environmental concerns and decarbonization

Anthropogenic factors are the fundamental cause behind global climate disruption and the emission of greenhouse gases (GHGs), which are responsible for disastrous consequences. These consequences include global warming, a decrease in Arctic sea and land ice, and a relative sea level rise, which have been observed during recent decades, endangering life on Earth. Consequently, various environmental directives have been implemented in an effort to eliminate adverse climatic factors and related consequences (Bompard et al. 2018; Rockström et al. 2017; Kopac 2023; Succar et al. 2023).

Efforts are increasing worldwide to significantly decrease GHG emissions as of 2050 in compliance with the Paris Agreement on Climate Change, which requires transformational measures (Sundaramoorthy et al. 2023; UNFCCC 2023). Specifically, regarding global warming, the importance of substantial GHG emission mitigation for decades to come has been highlighted, namely, to reach or approach near-zero levels by the close of the century, ensuring a temperature increase less than 2 °C relative to pre-industrial levels, particularly in an effort to constrain it to 1.5 °C by 2050 (Bompard et al. 2018; Hou et al. 2023; IPCC 2023; Rockström et al. 2017; Steffen et al. 2015; Succar et al. 2023). From 2017, the global mean temperature rise amounted to almost 1 °C and is estimated to reach the 1.5-°C indication as of 2040 in the case of these trends. A temperature limit of 2 °C implies attaining the zero emissions target in the latter half of this decade; however, when restricting the temperature to 1.5 °C, GHG emissions need to converge a nearly zero level as of 2050 (Rissman et al. 2020; Sundaramoorthy et al. 2023).

The decarbonization strategy encompasses various approaches, such as expanding the use of RESs; implementing carbon capture, utilization, and storage (CCUS) systems; promoting almost energy-efficient buildings; developing smart grids; implementing carbon pricing (taxing carbon emissions per ton of CO_2_); fostering CO_2_-free processes; and utilizing CO_2_ from external sources for the production of goods. These strategies play a pivotal role in reducing carbon emissions. The variety of RESs, including biomass, solar, wind, geothermal, and other emerging zero/low-carbon energy sources, is expected to expand (Bompard et al. 2018; Kopac 2021a; Chen et al. 2023; Nesterenko et al. 2023; Succar et al. 2023; Zheng and Jin 2023).

### Carbon capture technologies

It has been reported that nearly 79% of all CO_2_ emissions result from the fossil fuels combustion and the use of minerals for power production and that coal-powered plants play an important role in global warming (Aghaie et al. 2018; IPCC 2023; Kopac 2021b; Yu et al. 2008). CO_2_ emissions rely upon the content of the fuel (Madejski et al. 2022). Among the main constituents of flue gas resulting from fuel combustion, such as unburned fuel, CO_2_, N_2_O, N_2_, and SO_2_ currently exist largely in the atmosphere at continually rising levels exceeding 400 ppm (Varghese et al. 2020; Varghese and Karanikolos 2020). Hence, eliminating CO_2_ from flue gases is considered important for reducing carbon levels in the atmosphere. The utilization of fossil fuels, including coal, oil, and natural gas, obviously keeps going, so it would be appropriate to use them following the application of effective strategies for carbon mitigation (Aghaie et al. 2018). Many countries still use coal-powered systems due to their lower cost and safety, which cannot be completely substituted with RES operating systems (Madejski et al. 2022).

Carbon capture methods can be categorized into three primary groups: pre-combustion process, oxy-combustion, and post-combustion (Aghaie et al. 2018; Madejski et al. 2022; Arshad and Alhajaj 2023; Baudoux et al. 2024).

Pre-combustion methods are applied prior to combustion by means of gasification of fuel with oxygen, whereas oxy-combustion carbon capture methods are employed after combustion in the presence of oxygen by separating CO_2_ formed during oxy-combustion treatment. Post-combustion is used after combustion to capture CO_2_ from flue gas. This can be accomplished through diverse approaches, such as membrane separation, physical adsorption, chemical absorption, or chemical looping (Madejski et al. 2022). The main distinction between post-combustion and oxy-combustion methods lies in the composition of the flue gas. In oxy-combustion, the flue gas is intensely concentrated in CO_2_, making it appropriate for underground storage. Alternatively, the post-combustion technique requires additional procedures for carbon capture from flue gas (Aghaie et al. 2018; Chen et al. 2012). As the utilization of fossil fuels in current technologies continues until alternative fuels are replaced without CO_2_ emissions, CO_2_ capture is inevitable for reducing GHGs (Madejski et al. 2022).

The post-combustion technique for the capture of CO_2_ is more appropriate as an alternative for retrofitting in power plants than are the oxy-combustion and pre-combustion capture strategies, which can be implemented only in newly established power plants (Aghaie et al. 2018; Chen et al. 2012; Sreedhar et al. 2017a). However, the main barrier in the application of these methods is that since the CO_2_ partial pressure in flue gas is low, the driving force for CO_2_ would also be low (Wang et al. 2017). Post-combustion carbon capture methods depend on eliminating CO_2_ from flue gas, with the capturing unit installed following the purification system, such as desulfurization, denitrogenation, or dust removal units (Madejski et al. 2022).

Post-combustion technologies can be subdivided into several categories, such as absorption, microbial (algal), membrane or physical separation, adsorption, chemical looping for combustion, and cryogenic methods. Among those methods, absorption-based CO_2_ capture is among the commonly employed methods in commercial carbon capture plants due to its lower energy requirements and efficiency (Aghaie et al. 2018; Madejski et al. 2022; Mostafavi et al. 2021; Sreedhar et al. 2017a). Through this technique, the flue gas is treated by contacting it with a solvent in the absorption towers, which enables CO_2_ capture from the flue gas. The CO_2_ absorption rate of a solvent is an essential factor in the chemical absorption process. Higher absorption rates can result in capital cost savings for the process, as well as influencing the operation of the process at an industrial level (Aghaie et al. 2018; Sreedhar et al. 2017b).

### Amine-based CO_2_ capture

The physical absorption technique depends on the use of chemically inert solvents, such as organic solvents (methanol, N-methyl-2-pyrrolidone, or dimethyl ether), or simply water absorbing CO_2_ physically. Optimal results can be accomplished by operating at reduced temperatures and increased pressures for the separated gas (Madejski et al. 2022).

Chemical absorption is a well-known technique for CO_2_ capture based on reactions between CO_2_ and chemical solvents. This process has long been employed in chemical industries; however, it is energy intensive. Chemical absorption methods are utilized in solid fuel-fired power plants as the only selections available commercially. According to previous reports, amine methods possess the capability to capture approximately 85–95% of CO_2_ from flue gas at levels exceeding 99.95% purity (Madejski et al. 2022).

Different types of solvents for chemical absorption–based carbon capture have been used (Aghaie et al. 2018; Rochelle 2009). Among those, amine-based CO_2_ capture has found widespread industrial applications, including H_2_S removal, syngas purification, and various other processes, including those applied in the steel industry for decreasing large-scale CO_2_ emissions (Luo et al. 2016; Varghese and Karanikolos 2020; Ma’mun et al. 2007; Nielsen et al. 2012; Zhou et al. 2011). The amine-based carbon capture approach utilizes an amine solvent for the absorption of CO_2_ from flue gas. Subsequently, the CO_2_ is desorbed from the solvent, allowing for its reuse after regeneration (Zhou et al. 2011).

The solvents that are typically employed as absorbents for carbon capture operations include alkanolamines, including monoethanolamine (MEA) and diethanolamine (DEA); tertiary amines, such as MDEA; and sterically hindered amines, for example, AMP in aqueous solutions (Madejski et al. 2022), because of their strong reactivity toward CO_2_ molecules, significant absorption capacity, and temperature durability. Conversely, the process of amine-based carbon capture involves rather complicated work involving the follow-up of a number of operating variables, flow rate, pressure, temperature, and level of reaction instruments, as well as careful manipulation of numerous valves and pumps, directly impacting plant performance and CO_2_ capture efficiency (Zhou et al. 2011). Additionally, the application of amines has certain drawbacks, including equipment corrosion, high construction costs, and amine degradation by O_2_, NO_2_, and SO_2_ in flue gas. Consequently, absorbent regeneration processes at high temperatures require a significant amount of absorbent makeup and result in increased energy consumption. These drawbacks are correlated with the intrinsic features of amines, such as their corrosive nature, elevated vapor pressures, and substantial energy requirements for regeneration (Aghaie et al. 2018; Torralba-Calleja et al. 2013). Furthermore, conventional frameworks often suffer drawbacks, such as limited capacity to increase amine concentration, a highly diffusion-constrained process, and environmental pollution resulting from amine discharge (Nielsen et al. 2012).

Piperazine (PZ), MEA, and MDEA are among the widely employed amine solvents in industrial processes (Mostafavi et al. 2021). Moreover, lower energy expenditure was reported for PZ-AMP solvents and ammonia, which deserves further investigation (Madejski et al. 2022). Furthermore, MEA exhibits satisfactory absorption and desorption even when blended with different kinds of solvents (Chao et al. 2021). In addition to conventional solvents (MEA, DEA, PZ, ammonia), other solvents have been devised for CO_2_ capture processes. Blends of various solvents can potentially enhance absorptive properties through the use of proper combinations of various types of adsorbents. Primary and secondary amines indicate elevated absorption rates, while tertiary amines are identified by greater absorption (Madejski et al. 2022; Nord and Bolland 2020).

### Simulation studies on absorption-based CO_2_ capture

Reports show that the most appropriate solutions for existing coal-fired units are post-combustion methods (Madejski et al. 2022). Numerous investigations have been carried out to simulate the amine-based CO_2_ capture process. In general, the simulations involved a pre-treatment stage, comprising cooling down the flue gas by eliminating impurities involving particulate matter and other gaseous impurities, such as NO_*x*_ and SO_*x*_. After pre-treatment, the flue gas is injected into the absorber, where it is exposed to the lean solvent. Next, the solvent, rich in CO_2_, is introduced into the stripping column, where CO_2_ extraction from the solvent and regeneration of the lean solvent take place. The lean solvent is subsequently sent back to the absorbing column for further utilization in the procedure. Finally, the high-purity CO_2_ stream from the desorber can then be exposed to drying and post-treatment. It can be compressed and stored or subsequently employed (Madejski et al. 2022; Zhou et al. 2011).

Aspen Plus modeling of a post-combustion CO_2_ capture process for a coal-fueled power plant with PZ-enhanced K_2_CO_3_ solution (K_2_CO_3_/PZ) was studied by Oexmann et al. (2008). A sensitivity analysis was performed on the important operational parameters, including lean loading, solvent composition, CO_2_ capture rate, and desorption pressure, to identify optimal parameters that would minimize overall specific energy requirements. A stagewise equilibrium model was employed for modeling the absorber and desorber units, and the outcomes from these calculations were utilized to design the column sizing for each theoretical stage while accounting for kinetic effects. Analysis revealed significant energy savings by employing K_2_CO_3_/PZ with compression for the capture process in comparison to the conventional MEA process and lower investment costs because of the improved reaction kinetics, resulting in smaller component sizes.

Pellegrini et al. (2010) conducted an Aspen Plus modeling study to assess the CO_2_ capture efficiency of various solvents (MEA, DGA, and NH_3_) in a flue gas stream. The simulation used a conventional absorption–desorption unit configuration with RadFrac-type columns for chemical equilibrium calculations. The results indicated that ammonia was an effective absorbent, achieving effective removal efficiencies with minimal solvent use but required energy to reduce ammonia emissions.

Arachchige and Melaaen (2012) focused on simulating CO_2_ removal from flue gas streams for gas and coal fuel power plants using MEA solvent in Aspen Plus. With an 85% CO_2_ removal rate, the rate-based model utilized the electrolyte non-random two-liquid (ELECNRTL) method for simulating the reactive capture process and optimizing its settings. The reboiler duty decreased with increasing absorption column pressure and packing height, while the removal efficiency increased with increasing temperature and solvent concentration. Nevertheless, the efficiency of the removal process decreased with increasing lean loading of MEA solvent.

Jana and De (2014) proposed an integrated gasification combined cogeneration system utilizing sugarcane bagasse with CO_2_ capture using an aqueous MEA solution. This study utilized Aspen Plus for thermodynamic modeling, enabling a comparison between the pre- and post-combustion CO_2_ capture approaches. For the physical property determination of the conventional components involved in the system, the Peng–Robinson equation of state with an alpha function was utilized. This equation yields accurate results for correlating the vapor pressure of pure components at high temperatures due to the temperature-dependent variable, alpha. When estimating the thermo-physical properties of the process, the ELECNRTL property method demonstrated consistent reliability. The results indicated that the post-combustion capture process was the best pathway. Furthermore, the capture process had to be optimized based on the overall plant performance, as an increased capture level affects both the thermodynamic efficiency and cost-effectiveness of the system.

Lungkadee et al. (2021) carried out simulation studies for retrofitting a PCCC unit with a power plant (300 MW) using MEA as the solvent and reported the CO_2_ capture costs (≤ 55 $/ton). The absorber and stripping units were designed to have a 90% CO_2_ capture capacity with MEA (30 wt%). Approximately 63.075 kg/s of CO_2_ capture from flue gas (458 kg/s) was reported (Lungkadee et al. 2021). A simulation study of a natural gas combined cycle power plant utilizing PZ as a solvent demonstrated superior performance in comparison to MEA. Using PZ (40 wt%) exhibited noteworthy enhancements in capture effectiveness, energy utilization, and costs in comparison to employing MEA (30 wt%). The CO_2_ capture costs (min 34.65 $/ton) of the analysis with PZ solvent (40 wt%) were reported (Otitoju et al. 2021). El Hadri et al. (2017) studied various amine solutions (30 wt%) for PCCCs. Hexamethylenediamine showed the optimal CO_2_ loading, while triethanolamine showed the minimum CO_2_ loading.

Chuenphan et al. (2021) studied Aspen Plus modeling of CO_2_ capture using MEA with equilibrium-based methods and the ENRTL-RK thermodynamic property model. An experimental design involving a 2^k^ factorial methodology was implemented to examine the influence of several parameters, including the sour gas temperature, liquid-to-gas mass ratio, sour gas CO_2_ concentration, and lean MEA concentration and temperature, on CO_2_ capture and the reboiler heat duty. The study reported the optimization of these parameters for improving the process performance.

Sultan et al. (2021) evaluated the techno-economic analysis of the CO_2_ capture process for coal fuel power plants using an MEA solution. The conventional process and different stripper modifications (stripper overhead exchanger, lean vapor compression, and a hybrid configuration) were studied using the Aspen Plus rigorous rate-based model for simulating the absorber and stripper and the Aspen Process Economic Analyzer for optimizing the process. The ENRTL property model and Redlich–Kwong (RK) equation of state were applied for modeling the liquid (L) phase and vapor (V) phase, respectively. All of these modifications resulted in reduced energy consumption and demonstrated economic benefits. Using the optimal hybrid configuration (LVCSOE), both the solvent regeneration energy consumption and CO_2_ capture costs were reduced. An economic analysis revealed that, compared with other economic parameters, CO_2_ capture costs were most affected by regeneration steam costs.

### Aim of the present study

In our research, we focused on examining the significance of various thermodynamic models, as well as equilibrium- and rate-based models, to represent the reaction kinetics and the impact of different solvents in capturing CO_2_. While most studies in this field rely on established equilibrium modeling, our study delved into the importance of incorporating kinetic modeling and the specific role that different solvents play in the CO_2_ capture process. The novelty of the study lies in its comprehensive comparison of different amine solvents, evaluation of thermodynamic models, emphasis on rate-based modeling, and the analysis of CO_2_ inlet composition in capturing CO_2_. These aspects contribute to a deeper understanding of carbon capture processes and provide valuable insights for optimizing efficiency and for a further economic feasibility analysis.

The simulation studies were carried out for chemical absorption–based CO_2_ capture systems with different aqueous amine solvents, namely, AMP, DEA, and MDEA. In this framework, the performances of solvents, AMP, DEA, and MDEA were studied based on a flowsheet involving an absorber and stripper units. The impact of thermodynamics and kinetics on chemical absorption–based CO_2_ capture with respect to different amine solvents was investigated. For a steady evaluation of the efficacy of various amine solvents, the physical properties, kinetics, and thermodynamics need to be verified according to the requirements of Aspen Plus modeling. For the systems, parametric simulations were conducted to determine the impact of thermodynamics and kinetics on the carbon capture performance of the amine-based CO_2_ capture system.

## Materials and methods

The study utilizes a comprehensive methodology consisting of computer simulations, modeling, and data analysis to explore the decarbonization of flue gas through solvent-based CO_2_ capture. To achieve this, the following steps in the methodology were taken: a thorough literature review to establish the foundation and identify research gaps, utilization of Aspen Plus simulation software to model the carbon capture process, comparison of different amine solvents (AMP, DEA, and MDEA), use of thermodynamic methods (ELECNRTL, ENRTL-RK, ENRTL-HF, and ENRTL-HG), use of equilibrium- and rate-based models to evaluate solvent behavior and CO_2_ capture performance, analysis of mass flows to evaluate the performance of different solvents, calculation of CO_2_ capture percentage and sensitivity analysis to determine the impact of CO_2_ mole fraction in the feed gas on carbon capture performance, data analysis to compare results, identify trends, and draw conclusions. The methodology aims to provide insights into optimizing the carbon capture process by combining theoretical modeling, computer simulations, and data analysis.

### Modeling and simulation studies

#### Thermodynamic property models

In the simulations, the thermodynamic property methods and the reaction kinetic models utilized were obtained from the following sources: Aspentech (2014a) and Jamal et al. (2006a, b) for AMP; Aspentech (2014b) and Rinker et al. (1996) for DEA; and Aspentech (2020a), Austgen et al. (1991), Rinker et al. (1997), and Pinsent et al. (1956) for MDEA.

The thermodynamic models that were utilized in the simulations to depict the CO_2_ capture system with Aspen Plus modeling included true species, including ions, and electrolyte transport property models, which are described as follows.

ELECNRTL model: The electrolyte NRTL model, coupled with the RK equation of state, has been used for applications that involve aqueous and mixed solvents. This property model in Aspen Plus, which was first proposed by Chen et al. (1979) and Chen et al. (1982) and subsequently extended by Chen and Evans (1986) and Mock et al. (1986), can be used for determining the concentrations of aqueous and solvent mixtures. The model can be used for modeling the VLE of electrolyte systems and evaluating the excess Gibbs free energy of an electrolyte solution. The excess Gibbs free energy of the electrolyte system was modeled by combining short-range (ion-ion, molecule–molecule, local ion–molecule) and long-range (Pitzer–Debye–Huckel/Born) interactions (Chen et al. 1982; Chen and Evans 1986; Austgen 1989; Pitzer 1980; Robinson and Stokes 1970). The local interaction contributions are obtained in accordance with the NRTL model. The sole tunable model parameters involve the empirical binary energy interaction parameters and need to be evaluated via regression of available system data (Austgen 1989, Kothandaraman 2010).

ENRTL-RK model: This model combines the non-symmetric electrolyte NRTL model with the RK equation and Henry’s law which is suitable for electrolyte systems with a symmetric reference state for all components.

ENRTL-HF model: This model couples the electrolyte NRTL model with the HF equation of state which is ideal for mixed solvent applications.

ENRTL-HG model: This model utilizes the electrolyte NRTL model with the RK equation, incorporating the Helgeson model to estimate standard properties and equilibrium constants.

The equilibrium models as well as concentration-based reaction kinetics and rate-based models for the absorption and stripping columns involving packing were used in the simulations.

#### System overview

In the simulations for the CO_2_ capture processes by the three types of amine solvents (AMP/DEA/MDEA), the operational data of a pilot plant reported originally by Gabrielsen (2007; Aspentech 2014a) were utilized for the system composed of the absorber and stripper units. The typical sets of operation data employed in the simulations are presented in Table 1.

**Table 1:** Pilot plant data used in simulations (Gabrielsen 2007; Aspentech 2014a)

	Absorber	Stripper
Diameter	0.15 m	0.10 m
Type of packing	Sulzer Mellapak 250Y	
Height of packing	4.36 m	3.89 m
Feed and product streams		
Feed gas (sour) to absorber	118 m^3^/h	
Lean amine to absorber	3 L/min	
Rich amine to stripper	3 L/min	
CO_2_ in feed gas (sour)	0.130 (mole fraction)	
CO_2_ in sweet gas	0.098 (mole fraction)	
AMP solution	2.89 mol/L	

#### Physical properties

The abovementioned property models (ELECNRTL, ENRTL-RK, ENRTL-HF, and ENRTL-HG) and the equation of states were employed for computations of V and L properties in equilibrium- and rate-based models with the solvents AMP/DEA/MDEA.

Henry’s law was utilized for a selection of Henry components (solutes), including N_2_, O_2_, CO, H_2_, H_2_S, and CO_2_. Henry’s parameters for the components were determined using water and the solvents AMP/MDEA.

In the computations for the reaction, the basis selection for activity coefficients regarding Henry’s components was as aqueous. Hence, in determining the asymmetric activity coefficients of solutes, infinite dilution activity coefficients can be computed under conditions of unlimited dilution in pure water rather than in blended solvents.

Henry’s parameters were obtained from the Aspen Plus database for the components associated with water (Aspentech 2014a, b; Aspentech 2020a). Henry’s parameters for CO_2_ in water were estimated by regression from binary VLE data (Takenouchi and Kennedy 1964; Tödheide and Franck 1963; Dodds et al. 1956; Drummond 1981; Zawisza and Malesiska 1981; Wiebe and Gaddy 1940; Houghton et al. 1957). Henry’s constants of CO_2_ in AMP/MDEA solvents were acquired from the study conducted by Wang et al. (1992) (Aspentech 2014a; Aspentech 2020a). Henry’s constants in DEA were calculated by regression from CO_2_ solubility data (Maddox et al. 1987; Maddox and Elizondo 1989), H_2_S solubility data (Barreau et al. 2006), and literature (Lawson and Garst 1976) for H_2_S (Aspentech 2014b).

The NRTL interaction parameters for H_2_O-CO_2_ were assigned as zero, while those of H_2_O-AMP were calculated from regressions with binary VLE (Pappa et al. 2006), excess enthalpy (Mathonat et al. 1997), and heat capacity data (Chen and Li 2001; Chiu and Li 1999; Zhang et al. 2002). The NRTL interaction parameters for H_2_O-MDEA were determined via regression with binary VLE (Xu et al. 1991; Voutsas et al. 2004; Kim et al. 2008), excess enthalpy (Posey 1996; Maham et al. 1997, 2000), and heat capacity data (Chiu and Li 1999; Chen et al. 2001; Zhang et al. 2002). The NRTL parameters were calculated by regression of CO_2_ solubility (Maddox et al. 1987; Maddox and Elizondo 1989) and H_2_S solubility data for DEA (Barreau et al. 2006; Lawson and Garst 1976).

The interaction energy parameters for H_2_O-(AMPH^+^, HCO_3_^−^) were regressed from the CO_2_ heat of solution data in aqueous AMP (Arcis et al. 2007) and CO_2_ solubility data in aqueous AMP solution. The data (Robert and Mather 1988; Tontiwachwuthikul et al. 1991; Teng and Mather 1990; Li and Chang 1994; Seo and Hong 1992; Jane and Li 1997; Kundu et al. 2003) were used in the regression for CO_2_ solubility in AMP. The VLE data with high CO_2_ loading (loading > 1) were not included in the regression, so this model was applicable to VLE from low to moderate CO_2_ loading. The interaction energy parameters for H_2_O-(AMPH^+^, HS^−^) were obtained by regression utilizing H_2_S solubility data in aqueous AMP solution (Roberts and Mather 1988).

The interaction parameters for H_2_O-(MDEAH^+^, HCO_3_^*−*^), H_2_O-(MDEAH^+^, CO_3_^*−*2^), and MDEA-(MDEAH^+^, HCO_3_^−^) were calculated by regression from the ternary VLE data (Kuranov et al. 1996; Kamps et al. 2001; Ermatchkov et al. 2006; Jou et al. 1982, 1993a), CO_2_ absorption heat data (Mathonat 1995; Carson et al. 2000), ternary heat capacity data (Weiland et al. 1997), and MDEA-H_2_O-CO_2_ liquid-phase concentration data (Bottinger et al. 2008). The energy parameters for H_2_O-(MDEAH^+^, HS^*−*^) and MDEA-(MDEAH^+^, HS^−^) were estimated via regression from H_2_S solubility data in aqueous MDEA solution (Kuranov et al. 1996; Kamps et al. 2001; Huang and Ng 1998).

The calculation results of the transport and thermal properties and model parameters utilized in the simulations, such as the liquid viscosity, density, and thermal conductivity; surface tension (AMP-CO_2_-H_2_O; DEA-CO_2_-H_2_O; MDEA-CO_2_-H_2_O) (Weiland et al. 1998; Weiland 1996); liquid heat capacity (AMP-H_2_O; DEA-CO_2_-H_2_O; MDEA-CO_2_-H_2_O) (Chen and Li 2001; Weiland et al. 1997); excess enthalpy (AMP-H_2_O) (Mathonat et al. 1997); integral heat of solution (AMP-CO_2_-H_2_O; DEA-CO_2_-H_2_O; MDEA-CO_2_-H_2_O); VLE (AMP-H_2_O; MDEA-CO_2_-H_2_O) of the investigated systems; CO_2_ partial pressure (AMP-CO_2_-H_2_O; DEA-CO_2_-H_2_O); and H_2_S partial pressure (AMP-H_2_S-H_2_O; DEA-H_2_S-H_2_O), which were adapted according to the experimental data from the literature are summarized in Table 2.

**Table 2:** Various transport and thermal properties of the solvents

Properties	Reference
AMP	
Liquid density (AMP-CO_2_-H_2_O) (298.15 K)	Aspentech (2014a)
Liquid viscosity (AMP-CO_2_-H_2_O) (298.15 K)	Aspentech (2014a)
Surface tension (AMP-CO_2_-H_2_O) (298.15 K)	Aspentech (2014a)
Liquid thermal conductivity (AMP-CO_2_-H_2_O) (298.15 K)	Aspentech (2014a)
Liquid heat capacity (MP-H_2_O), experimental data	Chen and Li (2001); Aspentech (2014a)
Excess enthalpy (AMP-H_2_O) (308.15 K), experimental data	Mathonat et al. (1997); Aspentech (2014a)
Integral heat of solution of CO_2_ in aqueous AMP solution (322.5 K), experimental data	Arcis et al. (2007); Aspentech (2014a)
VLE (AMP-H_2_O), experimental data	Pappa et al. (2006); Aspentech (2014a)
CO_2_ partial pressure (AMP-CO_2_-H_2_O), experimental data	Roberts and Mather (1988); Tontiwachwuthikul et al. (1991); Aspentech (2014a)
H_2_S partial pressure (AMP-H_2_S-H_2_O), experimental data	Roberts and Mather (1988); Aspentech (2014a)
DEA	
Liquid density (DEA-CO_2_-H_2_O) (298.15 K), experimental data	Weiland et al. (1998); Aspentech (2014b)
Liquid viscosity (DEA-CO_2_-H_2_O) (298.15 K), experimental data	Weiland et al. (1998); Aspentech (2014b)
Surface tension (DEA-CO_2_-H_2_O) (298.15 K), experimental data	Weiland (1996)
Liquid thermal conductivity (DEA-CO_2_-H_2_O) (298.15 K)	Aspentech (2014b)
Liquid heat capacity (DEA-CO_2_-H_2_O) (298.15 K), experimental data	Weiland et al. (1997); Aspentech (2014b)
Heat of solution (DEA-CO_2_-H_2_O) (298.15 K), experimental data	Carson et al. (2000); Aspentech (2014b)
CO_2_ partial pressure (DEA-CO_2_-H_2_O), experimental data	Maddox and Elizondo (1989); Aspentech (2014b)
H_2_S partial pressure (DEA-H_2_S-H_2_O), experimental data	Lawson and Garst (1976); Aspentech (2014b)
MDEA	
Liquid density (MDEA-CO_2_-H_2_O) (298.15 K), experimental data	Weiland et al. (1998); Aspentech (2020a)
Liquid viscosity (MDEA-CO_2_-H_2_O) (298.15 K), experimental data	Weiland et al. (1998); Aspentech (2020a)
Surface tension (MDEA-CO_2_-H_2_O) (298.15 K), experimental data	Weiland (1996)
Liquid thermal conductivity (MDEA-CO_2_-H_2_O) (298.15 K)	Aspentech (2020a)
Liquid heat capacity (MDEA-CO_2_-H_2_O) (298.15 K), experimental data	Weiland et al. (1997); Aspentech (2020a)
Integral CO_2_ absorption heat in aqueous MDEA solution, experimental data	Mathonat (1995); Aspentech (2020a)
VLE of MDEA-CO_2_-H_2_O	Jou et al. (1982); Ermatchkov et al. (2006); Aspentech (2020a)

#### Reactions

AMP is a hindered primary amine where the amino group is linked to a tertiary carbon atom. DEA is a secondary amine with two ethanol groups connected to the nitrogen atom, and MDEA is a tertiary amine with a methyl group and two ethanol groups bonded to the nitrogen atom (Wang et al. 2004). AMP can react with H_3_O^+^ to form AMPH^+^. It may also associate with CO_2_ to form unstable carbamate, which readily reacts with other species in solution, ending with AMPH^+^. DEA can also react with H_3_O^+^ to form DEAH^+^ ions. It may also undergo a reaction with CO_2_ to form the carbamate ion DEACOO^−^. MDEA can react with H^+^ to form MDEAH^+^ ions. However, it is incapable of reacting with CO_2_ to generate carbamates, such as primary or secondary ethanolamines. The molecular, structural, and 3D images of AMP, DEA, and MDEA are shown in Table 3.

**Table 3:**
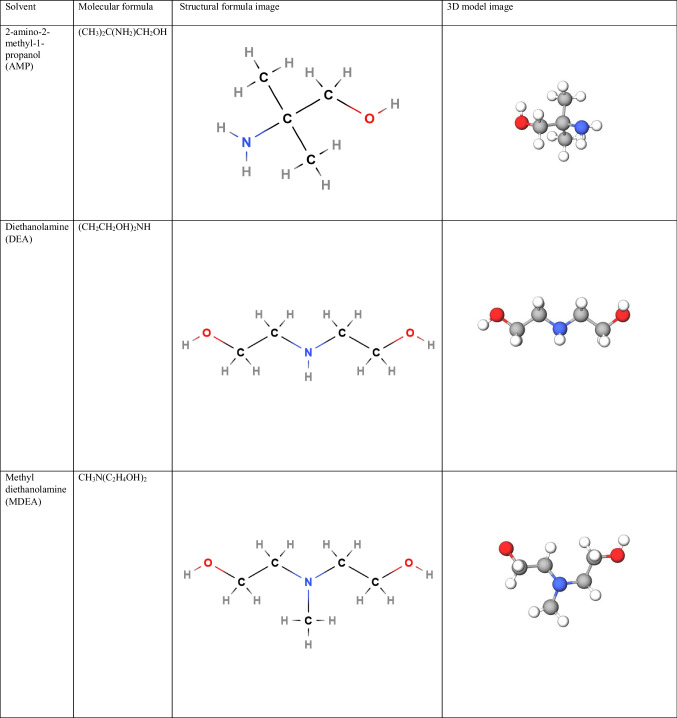
Amine-based solvents used for CO_2_ capture modeling: molecular, structural, and 3D images

The RadFrac distillation model in Aspen Plus12 offers both equilibrium- and rate-based modeling options that can be easily switched between two modes depending on the user selection used in the simulations. The equilibrium-stage approach for modeling distillation problems is among the most commonly used models in process simulators, as the solution algorithms are well-established and robust for a wide range of systems. Because of the ubiquity of equilibrium-based models, it is convenient to specify efficiency factors to introduce non-equilibrium behavior into equilibrium-stage models. For equilibrium-stage models, vaporization or Murphree efficiencies for stages or for column sections and component efficiencies for specific components in a given stage or column section can be specified. Although convenient, the use of equilibrium-stage models with vaporization and Murphree efficiencies has certain limitations, such as the variation in efficiency factors with column conditions (flow, temperature, pressure) and poor efficiency in reactive systems, as mass transfer due to kinetic or equilibrium reactions is not related to the phase equilibrium driving force and is also due to the requirement of numerous adjustable factors (Aspentech 2020b).

For the equilibrium-stage modeling approach in this present investigation, the electrolyte solution chemistries were simulated with CHEMISTRY Model identifiers, called CHEMISTRY ID = AMP, CHEMISTRY ID = DEA, and CHEMISTRY ID = MDEA. The CHEMISTRY IDs shown in Table 4 were used in the simulations. All the ionic reactions involved in the CHEMISTRY models identified by AMP, DEA, and MDEA given in Table 3 were assumed to be in chemical equilibrium.

**Table 4:** Reactions in equilibrium model

Reaction no	Type	Reaction chemistry
CHEMISTRY ID, AMP		
1	Equilibrium	2 H_2_O ⇔ H_3_O^+^ + OH^−^
2	Equilibrium	CO_2_ + 2 H_2_O ⇔ HCO_3_^−^ + H_3_O^+^
3	Equilibrium	HCO_3_^−^ + H_2_O ⇔ CO_3_^−2^ + H_3_O^+^
4	Equilibrium	AMPH^+^ + H_2_O ⇔ AMP + H_3_O^+^
5	Equilibrium	AMPCOO^−^ + H_2_O ⇔ AMP + HCO_3_^−^
6	Equilibrium	H_2_S + H_2_O ⇔ HS^−^ + H_3_O^+^
7	Equilibrium	HS^−^ + H_2_O ⇔ S^−2^ + H_3_O^+^
CHEMISTRY ID, DEA		
1	Equilibrium	DEAH^+^ + H_2_O ⇔ DEA + H_3_O^+^
2	Equilibrium	CO_2_ + 2 H_2_O ⇔ H_3_O^+^ + HCO_3_^−^
3	Equilibrium	HCO_3_^−^ + H_2_O ⇔ H_3_O^+^ + CO_3_^−2^
4	Equilibrium	DEACOO^−^ + H_2_O ⇔ DEA + HCO_3_^−^
5	Equilibrium	2 H_2_O ⇔ H_3_O^+^ + OH^−^
6	Equilibrium	H_2_S + H_2_O ⇔ HS^−^ + H_3_O^+^
7	Equilibrium	HS^−^ + H_2_O ⇔ S^−2^ + H_3_O^+^
CHEMISTRY ID, MDEA		
1	Equilibrium	2 H_2_O ⇔ H_3_O^+^ + OH^−^
2	Equilibrium	CO_2_ + 2 H_2_O ⇔ HCO_3_^−^ + H_3_O^+^
3	Equilibrium	HCO_3_^−^ + H_2_O ⇔ CO_3_^−2^ + H_3_O^+^
4	Equilibrium	MDEAH^+^ + H_2_O ⇔ MDEA + H_3_O^+^
5	Equilibrium	H_2_S + H_2_O ⇔ HS^−^ + H_3_O^+^
6	Equilibrium	HS^−^ + H_2_O ⇔ S^−2^ + H_3_O^+^

As an alternative to using vaporization and Murphree efficiencies with the equilibrium modeling approach, RadFrac also provides a rigorous rate-based modeling option that avoids some of the shortcomings linked to the efficiency approach (Aspentech 2020b). The rate-based model is particularly favorable for packed columns; absorption and desorption processes; reactive separation techniques involving reactive absorption and distillation, and strongly non-ideal systems; columns with complex configurations (such as those with pumparounds and side streams); and columns with both trays and packing (Aspentech 2017).

The rate-based feature of RadFrac in Aspen Plus enables rate-based simulation of absorption and stripping columns (Aspentech 2014a, b; Aspentech 2020a, b). The rate-based distillation approach utilizes heat and mass transfer correlations, adapted from the tray/packing geometry and the transfer properties, to analyze the column behavior. This eliminates the need for efficiency factors (Aspentech 2020b). It is based on various stages allowing the modeling of the kinetics of chemical reactions along with the heat and mass transfer phenomena involved. The several equations that can be solved include heat and mass balance equations for the L and V phases, heat and mass transfer rate models for calculating interphase transfer rates, VL equilibrium expressions for the interphase, calculations of heat and mass transfer coefficients and interfacial areas, and improvements in heat and mass transfer operations by chemical reactions (Aspentech 2008). The model solves the multicomponent Maxwell–Stefan mass transfer equation using two-film theory, in combination with the film and separate balance equations for the VL phases, reaction kinetics and diffusion, thermodynamics, and electrolyte solution chemistry. The hydrodynamics of the column are considered by using correlations for mass transfer coefficients, interfacial area, holdup, and pressure drop (Zhang et al. 2009; Kothandarama 2010; Aspentech 2017; Aspentech 2020b).

For the rate-based modeling approach in the present study, REACTION models called AMP-REA, DEA-REA, and MDEA-REA were established, as shown in Table 5; these models are used in the reaction calculations of the absorption and stripping columns. In the AMP-REA, DEA-REA, and MDEA-REA models, all of the reactions are considered to be in a state of chemical equilibrium except for the CO_2_-OH^*−*^ and CO_2_-AMP reactions and the CO_2_-DEA and CO_2_-MDEA reactions.

**Table 5:** Reactions in rate-based model

Reaction no	Type	Reaction chemistry
REACTION ID, AMP-REA		
1	Equilibrium	2 H_2_O ⇔ H_3_O^+^ + OH^−^
2	Equilibrium	HCO_3_^−^ + H_2_O ⇔ CO_3_^−2^ + H_3_O^+^
3	Equilibrium	AMPH^+^ + H_2_O ⇔ AMP + H_3_O^+^
4	Kinetic	CO_2_ + OH^−^ → HCO_3_^−^
5	Kinetic	HCO_3_^−^ → CO_2_ + OH^−^
6	Kinetic	AMP + CO_2_ + H_2_O → AMPCOO^−^ + H_3_O^+^
7	Kinetic	AMPCOO^−^ + H_3_O^+^ → AMP + CO_2_ + H_2_O
8	Equilibrium	H_2_S + H_2_O ⇔ HS^−^ + H_3_O^+^
9	Equilibrium	HS^−^ + H_2_O ⇔ S^−2^ + H_3_O^+^
REACTION ID, DEA-REA		
1	Equilibrium	DEAH^+^ + H_2_O ⇔ DEA + H_3_O^+^
2	Equilibrium	2 H_2_O ⇔ H_3_O^+^ + OH^−^
3	Equilibrium	HCO_3_^−^ + H_2_O ⇔ H_3_O^+^ + CO_3_^−2^
4	Kinetic	CO_2_ + OH^−^ → HCO_3_^−^
5	Kinetic	HCO_3_^−^ → CO_2_ + OH^−^
6	Kinetic	DEA + CO_2_ + H_2_O → DEACOO^−^ + H_3_O^+^
7	Kinetic	DEACOO^−^ + H_3_O^+^ → DEA + CO_2_ + H_2_O
8	Equilibrium	H_2_S + H_2_O ⇔ HS^−^ + H_3_O^+^
9	Equilibrium	HS^−^ + H_2_O ⇔ S^−2^ + H_3_O^+^
REACTION ID, MDEA-REA		
1	Equilibrium	MDEAH^+^ + H_2_O ⇔ MDEA + H_3_O^+^
2	Equilibrium	2 H_2_O ⇔ H_3_O^+^ + OH^−^
3	Equilibrium	HCO^−3^ + H_2_O ⇔ CO_3_^−2^ + H_3_O^+^
4	Kinetic	CO_2_ + OH^−^ → HCO_3_^−^
5	Kinetic	HCO_3_^−^ → CO_2_ + OH^−^
6	Equilibrium	H_2_S + H_2_O ⇔ HS^−^ + H_3_O^+^
7	Equilibrium	HS^−^ + H_2_O ⇔ S^−2^ + H_3_O^+^
8	Kinetic	MDEA + CO_2_ + H_2_O → MDEAH^+^ + HCO_3_^−^
9	Kinetic	MDEAH^+^ + HCO_3_^−^ → MDEA + CO_2_ + H_2_O

##### AMP

Equilibrium constants for reactions 1–5 in the AMP model were determined from the changes in the standard Gibbs free energy. The values for the aqueous phase free energy, heat of formation, and heat capacity at infinite dilution of AMPH^+^ and AMPCOO^*−*^ were utilized to calculate the standard Gibbs free energies of these components. The values for the other components, including the aqueous phase free energy, heat of formation, and heat capacity at infinite dilution, were extracted from the databank of Aspen Plus. Equilibrium constants for reactions 6–7 in the AMP model were sourced from the literature (Austgen et al. 1989).

The power law expression given by Eq. (1) was utilized for the rate-based reactions (reactions 4-7 of the AMP-REA model). In general, the mathematical expression for the power law can be given as:

1 $${\varvec{r}}={\varvec{k}}{\left(\frac{{\varvec{T}}}{{{\varvec{T}}}_{0}}\right)}^{{\varvec{n}}}{\varvec{e}}{\varvec{x}}{\varvec{p}}\left[\left(\frac{-{\varvec{E}}}{{\varvec{R}}}\right)\left(\frac{1}{{\varvec{T}}}-\frac{1}{{{\varvec{T}}}_{0}}\right)\right]\prod_{{\varvec{i}}=1}^{{\varvec{N}}}{{\varvec{C}}}_{{\varvec{i}}}^{{{\varvec{a}}}_{{\varvec{i}}}}$$

In Eq. (1), *r* is rate of reaction; *k* shows the pre-exponential factor; *T* corresponds to the temperature (absolute); *T*_0_ is the reference temperature; *E*, activation energy; *n*, exponent for temperature; *R*, gas constant; *C*_*i*_, concentration; *N*, number of components in the reaction; and *a*_*i*_, stoichiometric coefficient of components. When *T*_0_ is unspecific, the general power law expression given by Eq. (1) is reduced to the Eq. (2):

2 $${\varvec{r}}={\varvec{k}}{{\varvec{T}}}^{{\varvec{n}}}{\varvec{exp}}\left(\frac{-{\varvec{E}}}{{\varvec{R}}}\right)\prod_{{\varvec{i}}=1}^{{\varvec{N}}}{{\varvec{C}}}_{{\varvec{i}}}^{{{\varvec{a}}}_{{\varvec{i}}}}$$

In the present simulations, the reduced power law expression provided by Eq. (2) is utilized. The molarity basis of the concentration was used in this equation, and the exponent *n* = 0, pre-exponential factor *k*, and activation energy *E* are provided in Table 6. The kinetic constants for reaction 4 were extracted from the literature (Pinsent et al. 1956), while those of reaction 5 were computed according to the values of the kinetic constants of reaction 4 and the equilibrium constants of the equilibrium reactions 4 and 5. The second-order reaction kinetics expression for reaction 6 is used for the reaction between AMP and CO_2_, which is a simplification of the expression given by Jamal et al. (2006a, b). The kinetic parameters for reaction 6 given in Table 6 are those of *k*_1_ given by Jamal et al. (2006a, b), corresponding to the forward reaction rate constant for zwitterion formation.

**Table 6:** Kinetic parameters used in Eq. (2)

Reaction no	*k*	*E* (cal/mol)	Reference
AMP			
4	4.32e + 13	13,249	Pinsent et al. (1956); Aspentech (2014a)
5	2.38e + 17	29,451	Aspentech (2014a)
6	1.00e + 9	8202	Jamal et al. (2006a, b); Aspentech (2014a)
7	1.52e + 20	12,693	Aspentech (2014a)
DEA			
4	4.32e + 13	13,249	Pinsent et al. (1956); Aspentech (2014a, b)
5	2.38e + 17	29,451	Rinker et al. (1996); Aspentech (2014b)
6	6,480,000	5072	Rinker et al. (1996); Aspentech (2014b)
7	1.34e + 17	11,497	Rinker et al. (1996); Aspentech (2014b)
MDEA			
4	4.32e + 13	13,249	Pinsent et al. (1956); Aspentech (2014a, b); Aspentech (2020a)
5	2.38e + 17	29,451	Aspentech (2020a)
8	2.22e + 07	9029	Rinker et al. (1997); Aspentech (2020a)
9	1.06e + 16	25,424	Aspentech (2020a)

3 $${\varvec{A}}{\varvec{M}}{\varvec{P}}+{\varvec{C}}{{\varvec{O}}}_{2\boldsymbol{ }}\boldsymbol{ }\stackrel{{{\varvec{k}}}_{1}}{\to }\boldsymbol{ }{\varvec{A}}{\varvec{M}}{{\varvec{P}}}^{+}{\varvec{C}}{\varvec{O}}{{\varvec{O}}}^{-}$$

The kinetic parameters of reaction 7 were determined from the values of the kinetic parameters of reaction 6 and the equilibrium constants of reactions 6 and 7.

##### DEA

The equilibrium expressions for the reactions are obtained from the literature (Austgen et al. 1989; Jou et al. 1982; Jou et al. 1993a, b). The reduced form of the power law expression (Eq. 2) is employed for the kinetic reactions (reactions 4-7 of the DEA-REA model). The concentration was on a molarity basis, and the exponent *n* was taken as zero. The *k* and *E* values are tabulated in Table 6. The kinetic parameters for reactions 4-7 given in Table 5 are based on Rinker et al. (1996), provided that the ranges of DEA concentration (0.25–2.8 M) and temperature (292–343 K) correspond to the process conditions of the simulation system of the present study. The kinetic parameters for reaction 4 were sourced from the literature (Pinsent et al. 1956), while those of reaction 5 were computed utilizing the kinetic parameters of reaction 4 and the equilibrium constants of equilibrium reactions 4 and 5.

##### MDEA

Equilibrium constants for reactions 1–4 of the MDEA model were computed using the standard Gibbs free energy changes. The values of the aqueous phase free energy and heat of formation and the heat capacity at infinite dilution of MDEAH^+^ were determined and utilized to calculate the standard Gibbs free energy of MDEAH^+^. For the other components, the values of the parameters aqueous phase free energy and heat of formation and the heat capacity at infinite dilution were obtained from the software databank. The equilibrium constants for reactions 5–6 (MDEA model) were taken from the literature (Austgen et al. 1989). The reduced form of the power law equation was utilized for kinetic reactions 4, 5, 8, and 9 of the MDEA-REA model (Eq. 2). In the equation, the exponent *n* is zero, and the values of *k* and *E* are presented in Table 6. The kinetic parameters for reaction 4 given in Table 5 are obtained from the literature (Pinsent et al. 1956), while those of reaction 5 are computed by utilizing the kinetic parameters of reaction 4 and the equilibrium constants of the equilibrium reactions 4 and 5. The kinetic parameters for reaction 8 were extracted from Rinker et al. (1997), and those of reaction 9 were computed utilizing the kinetic parameters of reaction 8 and the equilibrium constants of equilibrium reactions 8 and 9.

#### Simulation approach

**Fig. 1:**
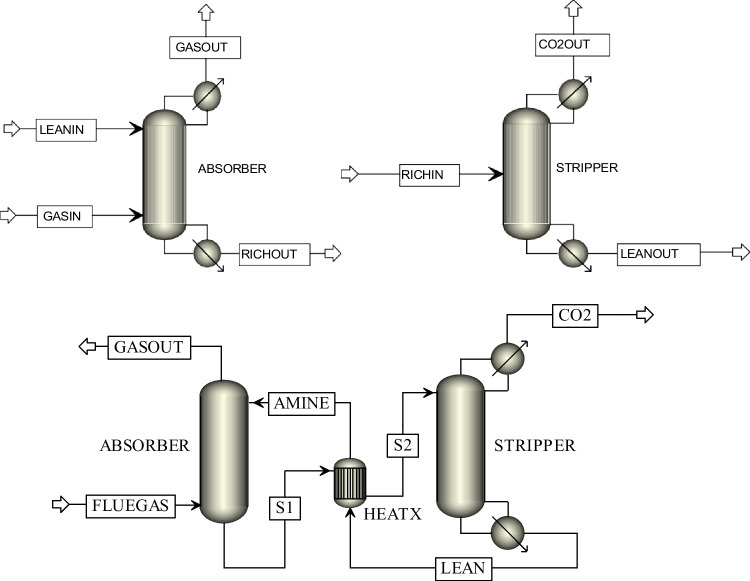
Schematic diagram of the absorption-based CO_2_ capture process involving absorber/stripper units used in simulations

Simulation studies were conducted utilizing experimental data from a pilot plant for CO_2_ absorption with AMP solution with structured packing in both absorption and stripping columns, as reported by Gabrielsen (2007). The modeling was carried out according to the simulation flowsheet in Aspen Plus, as indicated in Fig. 1. The major unit operations, as shown by the Aspen Plus blocks, and the specifications used in the modeling are described in Table 7. The correlations accounted for the heat (Chilton and Colburn 1934) and mass transfer coefficients, interfacial area (Bravo et al. 1985), and holdup (Bravo et al. 1992) utilized in modeling are also listed in the table.

**Table 7:** Configurations of absorber and stripper in the rate-based models

Unit operation, absorber (RadFrac)
• Rate-based, 11 stages • Top pressure, 1 atm • Reaction ID, AMP-REA/DEA-REA/MDEA-REA for all stages; for equilibrium calculations Holdup used, Holdup = 1.0e-5 kmol for convergence • Section diameter, 0.15 m
Unit operation, stripper (RadFrac)
• Rate-based, 9 stages • Partial vapor condenser, kettle reboiler • Mass distillate rate, 0.0018 kg/s • Mass reflux ratio, initialized at 0.3 with final value of 0.308 obtained by Design-Spec 1 • Top pressure, 1.95 bar, column pressure drop, 0.02 bar • Reaction: Chemistry ID, AMP/DEA/MDEA for stages 1 and 9; Reaction ID, AMP-REA/DEA-REA/MDEA-REA for stages 2–8; for equilibrium calculations Holdup used, Holdup = 0.00015 m^3^ for convergence • Design specs, stage 1 temperature = 292.15 K • Section diameter, 0.1 m
Common specifications for absorber/stripper units
• Packing, 250Y Standard Mellapak (Sulzer) • Mass transfer coefficient; interfacial area (Bravo et al. 1985); interfacial area factor, 1 • Heat transfer coefficient (Chilton and Colburn 1934) • Holdup correlation (Bravo et al. 1992); holdup scale factor, 1 • Film resistance, Discrxn for liquid film, Film for vapor film • Additional discretization points for liquid film, 5 • Flow model, mixed

The feed specifications and feed stream conditions for the absorption and stripping columns are supplied in Table 8. GASIN is the feed entering the absorber and contains N_2_, CO_2_, and H_2_O. LEANIN is the liquid lean solvent stream to the absorber containing aqueous AMP/DEA/MDEA solution. The feed to the stripper is a rich solvent stream, RICHIN, that contains aqueous AMP/DEA/MDEA solution with absorbed CO_2_.

**Table 8:** Stream results

Streams	GASIN	LEANIN	RICHIN
Temperature	41 °C	41 °C	41 °C
Pressure	1 atm	1 atm	1.96 bar
Total flow	118 m^3^/h	3 L/min	3 L/min
	Mole fraction	Molar concentration, mol/L	
AMP/DEA/MDEA	0	2.89	2.89
H_2_O	0.078	Solvent	Solvent
CO_2_	0.129	0.491	1.327
N_2_	0.793	0	0
Total	1.0	3.381	4.217

## Results and discussion

### Effect of solvent type, thermodynamics models, and rate versus equilibrium models on carbon capture

Traditionally, absorption and desorption columns can be modeled through equilibrium models in which the columns are segmented into several stages under the assumption that the L and V phases departing from a stage in a column are in a state of equilibrium (Treybal 1981). Nevertheless, this oversimplified assumption is mostly invalid in real instances. Therefore, corrections need to be made by employing parameters such as stage and Murphee efficiencies and the height equivalent to a theoretical plate (Taylor et al. 2003). However, specifically for reactive separation operations, the utilization of efficiency factors does not occur correctly as the discrepancies from the equilibrium models become too large (Aspiron 2006). Therefore, under such circumstances, the implementation of rate-based models for modeling systems is required (Kothandarama 2010). The rate-based modeling approach offers several advantages in comparison to the equilibrium-stage modeling. These models offer a more precise representation of the system and yield more realistic simulation results than equilibrium-stage models. This improved accuracy reduces the risk of inadequate designs or deviations from desired operating conditions. Rate-based models explicitly consider the specific column configuration, which directly influences the column efficiency. Although the rate-based model is more complex as compared to the equilibrium model, its complexity is hidden in the background. However, configuring a RateSep simulation is easy for users. RateSep includes several embedded correlations for holdup and mass transfer, supports film reactions and film discretization, and can accurately solve reactive separation problems. The application of the model can be extended to model novel situations with greater accuracy (Aspentech 2017).

The rate-based approach considers that separation occurs due to mass transfer between the interacting phases. The Maxwell–Stefan theory is utilized for calculating the rates of mass transfer. Conversely, the equilibrium model takes into account the equilibrium of the contacting phases. However, this assumption is critically flawed as the contacting phases could never be in equilibrium in real columns (Aspentech 2017).

As per the literature, the rate-based model is generally considered to produce more reliable results than the equilibrium model when applied to empirical data in reactive separation systems. Therefore, for reliable system simulations, the rate-based model ought to be the preferred option for reactive separation processes (Eckert and Vanek 2001; Springer et al. 2002; Taylor et al. 2003; Aspentech 2017). For instance, Kenig et al. (2001) reported that the rate-based modeling approach accurately predicts concentration profiles in H_2_S scrubbers, whereas the equilibrium model may yield inconsistent results compared to those of experimental studies. Eckert and Vanek (2001) reported that the rate-based model outperforms the equilibrium model in predicting concentration profiles, particularly in the middle of three-phase distillation columns. In the modeling of sour gas absorption by aqueous amine solutions, the rate-based model precisely predicts both composition and temperature profiles (Kucka et al. 2003). Klöker et al. (2005) employed rate-based and equilibrium models for reactive separation processes and discovered that the rate-based model, with fine discretization, better matches the column bottom concentration and temperature profile. The rate-based modeling approach has proven successful in modeling numerous industrial separation processes, including reactive distillation (Klöker et al. 2005; Dhale et al. 2004), reactive absorption (Kucka et al. 2003; Klöker et al. 2005; Bolhàr-Nordenkampf et al. 2004), reactive stripping (Klöker et al. 2005), and three-phase distillation (Repke et al. 2004; Higler et al. 2004).

In this study, the simulations were conducted using Aspen Plus12. The critical simulation findings are outlined in Tables 9, 10 and 11 for the solvents AMP, DEA, and MDEA, respectively. A comparison of the results for the ELECNRTL, ENRTL-RK, ENRTL-HF, and ENRTL-HG thermodynamic models for both the rate and equilibrium models is presented for each of the solvents in the tables. For the absorber and stripper units, the total mass flow rates and the CO_2_ mass flow rates in kilograms per hour corresponding to GASIN and GASOUT are shown.

**Table 9:** Carbon capture results for AMP

AMP			Mass flows (kg/h)							
Models			Absorber					Stripper		
			GASIN	GASOUT	LEANIN	RICHOUT	CO_2_ captured %	RICHIN	LEANOUT	CO_2_OUT
ELECNRTL	RATE	Total flow	134.31	130.98	180.99	184.32		178.81	172.33	6.48
		CO_2_	26.03	24.43	0.001	0.009	90.82	0.062	0.032	6.45
	EQM	Total flow	134.31	133.81	180.99	181.49		178.81	172.33	6.48
		CO_2_	26.03	25.57	0.001	0.025	96.66	0.062	0.032	6.45
ENRTL-RK	RATE	Total flow	134.31	131.34	178.15	181.12		175.70	169.22	6.48
		CO_2_	26.03	21.25	0.002	0.045	95.51	1.42	0.030	6.45
	EQM	Total flow	134.31	133.84	178.15	178.62		175.70	169.22	6.48
		CO_2_	26.03	25.48	0.002	0.044	95.43	1.42	0.030	6.45
ENRTL-HF	RATE	Total flow	134.23	130.81	178.75	182.17		177.21	170.73	6.48
		CO_2_	26.03	20.32	0.001	0.010	91.56	0.062	0.032	6.45
	EQM	Total flow	134.23	133.68	178.75	179.31		177.21	170.73	6.48
		CO_2_	26.03	25.56	0.001	0.025	96.66	0.062	0.032	6.45
ENRTL-HG	RATE	Total flow	134.31	130.27	190.05	194.10		201.64	195.16	6.48
		CO_2_	26.03	19.35	0.001	0.010	92.34	0.048	0.029	6.45
	EQM	Total flow	134.31	133.77	190.05	190.60		201.64	195.16	6.48
		CO_2_	26.03	25.49	0.001	0.029	97.21	0.048	0.029	6.45

**Table 10:** Carbon capture results for DEA

DEA			Mass flows (kg/h)							
Models			Absorber					Stripper		
			GASIN	GASOUT	LEANIN	RICHOUT	CO_2_ captured %	RICHIN	LEANOUT	CO_2_OUT
ELECNRTL	RATE	Total flow	134.31	130.25	187.26	191.32		186.96	180.48	6.48
		CO_2_	26.03	18.29	0.0002	0.012	98.23	0.131	0.031	6.48
	EQM	Total flow	134.35	131.37	188.68	191.66		194.38	187.90	6.48
		CO_2_	26.03	22.53	0.001	0.015	92.11	0.261	0.052	6.48
ENRTL-RK	RATE	Total flow	134.31	130.07	187.43	191.67		186.96	180.48	6.48
		CO_2_	26.03	17.90	0.00004	0.096	99.95	0.131	0.031	6.48
	EQM	Total flow	134.31	132.21	187.43	189.54		186.96	180.48	6.48
		CO_2_	26.03	21.58	0.00004	0.133	99.97	0.131	0.031	6.48
ENRTL-HF	RATE	Total flow	134.23	130.12	187.41	191.52		186.96	180.48	6.48
		CO_2_	26.01	18.25	0.0002	0.012	98.29	0.131	0.031	6.48
	EQM	Total flow	134.23	132.10	187.41	189.54		186.96	180.48	6.48
		CO_2_	26.01	21.74	0.0002	0.013	98.39	0.131	0.031	6.478
ENRTL-HG	RATE	Total flow	134.31	129.44	201.94	206.81		186.96	180.48	6.48
		CO_2_	26.03	17.11	0.0002	0.015	98.46	0.131	0.031	6.48
	EQM	Total flow	134.31	132.14	201.94	204.12		186.96	180.48	6.48
		CO_2_	26.03	21.59	0.0002	0.016	98.56	0.131	0.031	6.48

**Table 11:** Carbon capture results for MDEA

MDEA			Mass flows (kg/h)							
Models			Absorber					Stripper		
			GASIN	GASOUT	LEANIN	RICHOUT	CO_2_ captured %	RICHIN	LEANOUT	CO_2_OUT
ELECNRTL	RATE	Total flow	134.31	131.89	186.48	188.89		187.22	180.74	6.48
		CO_2_	26.03	21.81	0.004	0.012	67.88	0.377	0.060	6.48
	EQM	Total flow	134.31	133.42	186.48	187.37		187.22	180.74	6.48
		CO_2_	26.03	24.94	0.004	0.012	81.24	0.377	0.060	6.48
ENRTL-RK	RATE	Total flow	134.31	131.03	186.44	189.73		187.22	180.74	6.48
		CO_2_	26.03	21.14	0.003	0.014	76.97	0.377	0.060	6.48
	EQM	Total flow	134.31	133.29	186.44	187.47		187.22	180.74	6.48
		CO_2_	26.03	24.96	0.003	0.019	83.63	0.377	0.060	6.48
ENRTL-HF	RATE	Total flow	134.31	131.60	186.77	189.41		187.22	180.74	6.48
		CO_2_	26.01	21.38	0.004	0.013	71.97	0.377	0.060	6.48
	EQM	Total flow	134.31	133.29	186.77	187.71		187.22	180.74	6.48
		CO_2_	26.01	24.92	0.004	0.020	81.40	0.377	0.060	6.48
ENRTL-HG	RATE	Total flow	134.31	131.25	195.71	198.77		187.22	180.74	6.48
		CO_2_	26.03	20.71	0.004	0.017	77.22	0.377	0.060	6.48
	EQM	Total flow	134.31	133.29	195.71	196.74		187.22	180.74	6.48
		CO_2_	26.03	24.73	0.004	0.023	83.70	0.377	0.060	6.48

For the assessment of CO_2_ emission levels and to measure the ability of CO_2_ capture to control emissions, a number of indicators, depending on the choice, can be used (Manzolini et al. 2020; Voldsund et al. 2019). The most widely used ones are the specific emission of CO_2_, relative emissivity of CO_2_, CO_2_ capture ratio, CO_2_ emission index, CO_2_ captured, CO_2_ avoided, CO_2_ emitted, specific primary energy consumption cost for prevented CO_2_, and levelized costs of electricity (Madejski et al. 2022; Nord and Bolland 2020).

In this study, CO_2_ captured was used as an indicator for the assessment of CO_2_ emissions. The CO_2_ captured percentage (%) in the absorber unit was calculated as follows:

4 $${\mathrm{CO}}_2\;\mathrm{captured}\;\%\;\mathrm{in}\;\mathrm{the}\;\mathrm{absorber}=\frac{\boldsymbol C{\boldsymbol O}_{\mathbf2}\;\left(\boldsymbol R\boldsymbol I\boldsymbol C\boldsymbol H\boldsymbol O\boldsymbol U\boldsymbol T\boldsymbol-\boldsymbol L\boldsymbol E\boldsymbol A\boldsymbol N\boldsymbol I\boldsymbol N\right)}{\boldsymbol C{\boldsymbol O}_{\mathbf2}\;\left(\boldsymbol R\boldsymbol I\boldsymbol C\boldsymbol H\boldsymbol O\boldsymbol U\boldsymbol T\right)}\times\mathbf{100}$$

Table 9 presents simulation results for the AMP solvent. When the ELECNRTL thermodynamic model was used with the rate-based model, CO_2_ capture was found to be 90.82%, while it was 96.66% for the equilibrium model, indicating a discrepancy of 6.43% between the results of the models, evaluated as the absolute value of the difference between the CO_2_ capture from the equilibrium model and the CO_2_ capture from the rate-based model, divided by the CO_2_ capture from the rate-based model. Similarly, for the rate-based model, when the ENRTL-RK thermodynamic model was used, the CO_2_ capture rate was determined to be 95.51%, whereas it was 95.43% for the equilibrium model, indicating a discrepancy of only 0.08%. For the rate-based model with the ENRTL-HF thermodynamic model, CO_2_ capture was 91.56%, whereas it was 96.66% for the equilibrium model, indicating a discrepancy of 5.57%. Finally, for the rate-based model with the ENRTL-HG thermodynamic model, CO_2_ capture was 92.34%, whereas it was 97.21% for the equilibrium model, indicating a discrepancy of 5.27%. Table 10 summarizes the results for DEA. When the ELECNRTL thermodynamic model was used with the rate-based model, CO_2_ capture was found to be 98.23%, while for the equilibrium model, it was 92.11%, indicating a discrepancy of 6.23%. Similarly, for the rate-based model, when the ENRTL-RK thermodynamic model was used, the CO_2_ capture rate was determined to be 99.95%, whereas it was 99.97% for the equilibrium model, indicating a discrepancy of just 0.68%. For the rate-based model with the ENRTL-HF thermodynamic model, CO_2_ capture was 98.29%, whereas it was 98.39% for the equilibrium model, indicating a discrepancy of just 0.09%. Finally, for the rate-based model with the ENRTL-HG thermodynamic model, CO_2_ capture was 98.46%, whereas it was 98.56% for the equilibrium model, indicating a discrepancy of just 0.1%. Table 11 presents the results for MDEA. CO_2_ capture was found to be 67.88% for the rate-based model with ELECNRTL thermodynamic model, whereas it was 81.24% for the equilibrium model. This indicates a discrepancy of 19.68%. Similarly, for the rate-based model with the ENRTL-RK thermodynamic model, CO_2_ capture was found to be 76.97%, whereas it was 83.63% for the equilibrium model, indicating a discrepancy of 8.65%. For the rate-based model with the ENRTL-HF thermodynamic model, CO_2_ capture was found to be 71.97%, whereas it was 81.40% for the equilibrium model, indicating a discrepancy of 13.10%. Finally, for the rate-based model with the ENRTL-HG thermodynamic model, CO_2_ capture was 77.22%, whereas it was 83.70% for the equilibrium model, indicating a discrepancy of 8.39%.

**Fig. 2:**
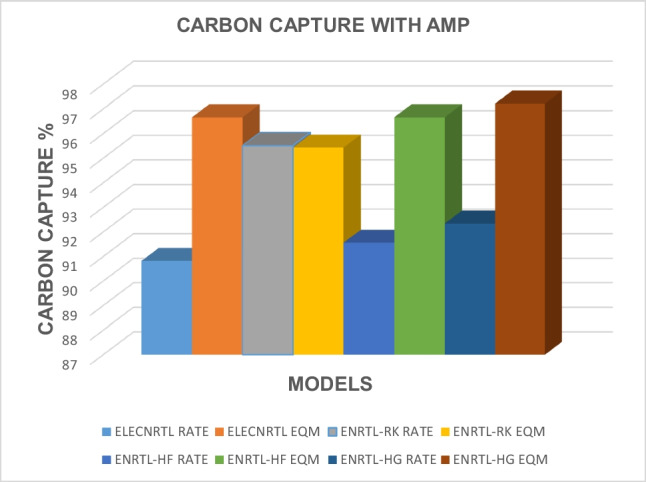
Carbon capture results obtained with AMP for different thermodynamic property models and equilibrium- and rate-based models

**Fig. 3:**
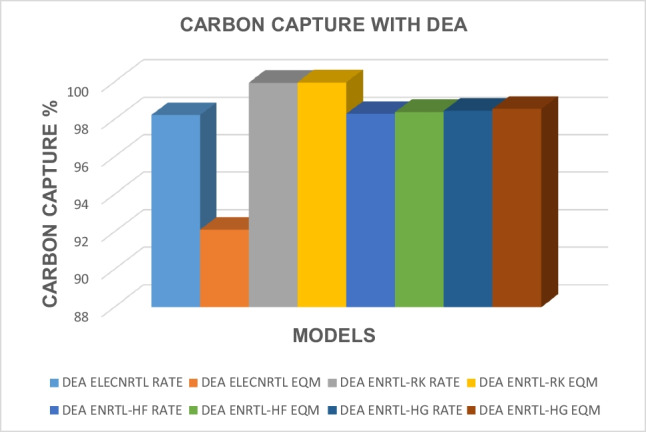
Carbon capture results obtained with DEA for different thermodynamic property models and equilibrium- and rate-based models

**Fig. 4:**
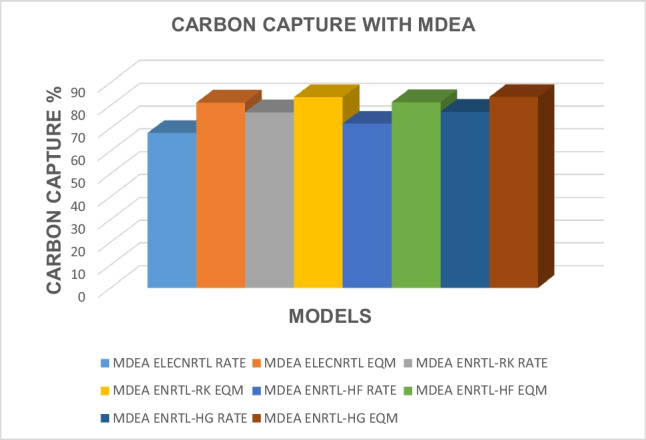
Carbon capture results obtained with MDEA for different thermodynamic property models and equilibrium- and rate-based models

The impact of the type of solvent used on carbon capture has been analyzed and the results for all the models corresponding to all the solvents are presented in Table 12. The carbon capture results obtained with AMP for different thermodynamic, rate, and equilibrium models are presented in Fig. 2. Figure 3 shows the results for carbon capture with DEA solvent for the different thermodynamic models and rate and equilibrium models, and Fig. 4 shows the results for the solvent MDEA. It is evident that for the solvent AMP, higher CO_2_ capture results in the absorber were obtained for the equilibrium models when the ELECNRTL, ENRTL-HF, and the ENRTL-HG thermodynamic models were used, while ENRTL-RK gave similar CO_2_ capture results for both the rate and the equilibrium models. For the MDEA solvent, higher CO_2_ capture results in the absorber were obtained for the equilibrium models when the ELECNRTL, ENRTL-RK, ENRTL-HF, and ENRTL-HG thermodynamic models were used. It is worth noting that traditional models assume that the absorption and desorption columns are in a state of equilibrium, in which the columns are segmented into several stages assuming that the L and V phases departing from one stage in the column are in a state of equilibrium. However, this oversimplified assumption is mostly inaccurate in real instances; specifically, for reactive separation operations, the discrepancies from the equilibrium models become too large. Therefore, applying rate-based models for modeling the systems is needed under such circumstances.

**Table 12:** Carbon capture (%) with the solvents AMP/DEA/MDEA and thermodynamic property models

ELECNRTL		ENRTL-RK		ENRTL-HF		ENRTL-HG	
Rate	EQM	Rate	EQM	Rate	EQM	Rate	EQM
AMP							
90.82	96.66	95.51	95.43	91.56	96.66	92.34	97.21
DEA							
98.23	92.11	99.95	99.97	98.29	98.39	98.46	98.56
MDEA							
67.88	81.24	76.97	83.63	71.97	81.4	77.22	83.70

For solvent DEA, higher CO_2_ capture results in the absorber were acquired for the rate-based models when the ELECNRTL thermodynamic model was applied, while the ENRTL-HF, the ENRTL-HG, and the ENRTL-RK gave similar CO_2_ capture values for both the equilibrium and rate models. It has been reported in the literature that secondary amines exhibit less corrosiveness and demand less heat for regeneration. This is attributed to the additional ethanol group, which, when compared to primary amines, diverts a significant portion of the free electron character away from the nitrogen atom (Wang et al. 2004; Gunasekaran et al. 2013).

It is evident that the lowest carbon capture values for the absorber unit were obtained with the solvent MDEA. Higher carbon capture values were obtained with the AMP solvent than with MDEA. The optimal outcomes were achieved with the solvent DEA for all the thermodynamic models applied, with similar results for both the rate and the equilibrium models.

According to the simulation results, the solvents can be ranked in terms of their carbon capture performance, with DEA being the most effective, followed by AMP and MDEA. The lowest carbon capture values were obtained with the MDEA solvent. In comparison, higher carbon capture values were obtained with the AMP solvent than MDEA. However, the optimal outcomes were achieved with the DEA solvent for all thermodynamic property models applied. Both the rate and equilibrium models produced similar results. In fact, the best results were obtained with the DEA solvent when using the rate-based model with the ENRTL-RK thermodynamic property model. This produced a carbon capture value of 99.95%, with a very low discrepancy of only 0.68% when compared to the equilibrium model. For the AMP solvent, the rate-based model with the ENRTL-RK thermodynamic model produced a CO_2_ capture rate of 95.51%, compared to 95.43% with the equilibrium model, indicating a discrepancy of only 0.08%. DEA has several advantages, such as low vapor pressure, minimal reactivity with COS and CS_2_, and low corrosiveness compared to primary amines. However, it does have some drawbacks, including difficulty reclaiming contaminants, inadequate treatment of gas streams with high CO_2_ levels, and the formation of corrosive degradation products when combined with CO_2_ (Luo et al. 2016). On the other hand, AMP, a hindered amine, has decent performance and some advantages, such as ease of regeneration compared to MEA, high CO_2_ loading, excellent CO_2_ absorption, higher degradation resistance, and low corrosion rates. However, it also has some disadvantages, such as lower CO_2_-amine mass transfer rates than MEA and larger substituents that cause its carbamate to be unstable, making it easy to form a bicarbonate. The MDEA solvent had the lowest CO_2_ capture performance based on simulation results. However, it does have some advantages, such as selective capture of H_2_S in the presence of CO_2_, good performance in concentrations up to 60% in aqueous solutions, resistance to degradation, low corrosiveness, low specific heat and heat of reaction with H_2_S and CO_2_, and thin miscibility with hydrocarbons. Additionally, it has a better distribution of driving force due to the nature of the gas–liquid equilibria, and it does not react with COS and CS_2_, resulting in low solvent loss. However, it does have a disadvantage in that it has a lower heat of reaction and a slow reaction with CO_2_. According to a prior experimental study by Luo et al. (2016), a fast screening method was tested on amine-based solvents for post-combustion CO_2_ capture. The study examined the effectiveness of single amines, including MEA, MDEA, AMP, and DEA, in aqueous solutions. The findings indicated that AMP and DEA outperformed MDEA in terms of CO_2_ capture. These results were further confirmed by Aspen Plus process simulations conducted under identical experimental conditions for each of the amines (Luo et al. 2016). Despite the outstanding CO_2_ capture performance of certain amines, selecting the appropriate solvent system remains a costly and time-consuming process, considering all aspects.

### Sensitivity analysis for determining the effect of the GASIN CO_2_ composition

**Fig. 5:**
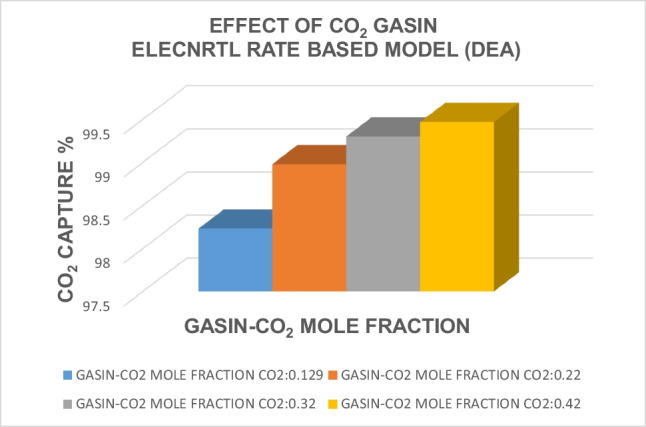
Effect of CO_2_ feed gas composition on carbon capture with DEA for the thermodynamic model ELECNRTL and rate-based models

Based on previous calculations, the CO_2_ mole fraction in GASIN was selected to be 0.129. In order to assess the influence of the CO_2_ mole fraction on CO_2_ capture, the calculations were repeated for different values of the CO_2_ mole fraction in GASIN, specifically 0.22, 0.32, and 0.42, using both the solvent DEA and the ELECNRTL rate-based model. Table 13 displays the impact of the CO_2_ inlet composition (GASIN) on the ELECNRTL rate-based model for the solvent DEA. Additionally, Table 14 provides a comparative analysis of the effect of the CO_2_ inlet composition (GASIN) on the final carbon capture in the absorber unit for the ELECNRTL rate-based model using the DEA solvent. Furthermore, Fig. 5 visually represents the influence of the CO_2_ feed gas composition on carbon capture with DEA for the ELECNRTL and rate-based models. The figure highlights the favorable impact of the CO_2_ composition in GASIN, demonstrating that higher carbon capture results can be achieved as the CO_2_ mole fraction in GASIN increases.

**Table 13:** Effect of CO_2_ inlet composition (GASIN) with thermodynamic property model ELECNRTL and rate-based model

DEA		Mass flows (kg/h)							
CO_2_ inlet composition (mole fraction)		Absorber					Stripper		
		GASIN	GASOUT	LEANIN	RICHOUT	CO_2_ captured %	RICHIN	LEANOUT	CO_2_OUT
0.129	Total flow	134.31	130.25	187.26	191.32		186.96	180.48	6.48
	CO_2_	26.03	18.29	0.0002	0.012	98.23	0.131	0.031	6.48
0.22	Total flow	141.03	136.34	187.26	191.95		186.96	180.48	6.48
	CO_2_	44.40	35.13	0.0002	0.020	98.98	0.131	0.031	6.48
0.32	Total flow	148.42	143.23	187.26	192.38		186.96	180.48	6.48
	CO_2_	64.61	54.29	0.0002	0.029	99.30	0.131	0.031	6.48
0.42	Total flow	155.81	150.38	187.26	192.69		186.96	180.48	6.48
	CO_2_	84.83	73.78	0.0002	0.039	99.47	0.131	0.031	6.48

**Table 14:** Effect of CO_2_ inlet composition (GASIN) with ELECNRTL and rate-based model with solvent DEA

CO_2_ inlet composition (mole fraction)	CO_2_ captured %
0.129	98.23
0.22	98.98
0.32	99.30
0.42	99.47

## Conclusions and future perspectives

In this study, the process of CO_2_ capture from a coal-fired power plant flue gas involving absorption and stripping units has been investigated using Aspen Plus equilibrium- and rate-based models, as well as various thermodynamic models, to examine the effectiveness of the aqueous amine solvents AMP, DEA, and MDEA in capturing CO_2_. The study utilized a comprehensive methodology consisting of computer simulations, modeling, and data analysis to explore the decarbonization of flue gas through solvent-based CO_2_ capture.

The simulation results show that DEA is the most effective solvent for carbon capture, followed by AMP and MDEA. The lowest carbon capture values were obtained with the MDEA solvent. The best results were achieved with the DEA solvent for all thermodynamic property models applied. The rate-based model with the ENRTL-RK thermodynamic property model produced the optimal outcomes for both DEA (99.95%) and AMP solvents (95.51%), with very low discrepancies in comparison to the equilibrium model. The study’s findings show a positive effect of the inlet CO_2_ composition, indicating that higher levels of carbon capture can be attained as the concentration of CO_2_ in the inlet increases.

The rate-based AMP-DEA-MDEA models provided a rate-based, rigorous simulation of the chemical absorption–based CO_2_ capture process. The notable features of the simulation included solution chemistry and electrolyte thermodynamics, rigorous transport property modeling, liquid-phase reaction kinetics, and rate-based multistage simulation with Aspen rate-based distillation incorporating heat and mass transfer correlations, taking into consideration the details of the absorbing and stripping columns and hydraulic characteristics. The methodology provided insights into optimizing the carbon capture process by combining theoretical modeling, computer simulations, and data analysis.

The model can be applied for modeling the CO_2_ capture process with different solvents other than AMP, DEA, and MDEA solvents and various blends of solvents. These studies can be extended to evaluate the cost of CO_2_ capture systems using diverse solvent systems.

These processes can be analyzed further, specifically by examining how essential operational variables such as solvent loading and composition, desorption pressure, and the CO_2_ capture rate affect the energy cost and the overall cost of capturing CO_2_ (expressed in cost/ton CO_2_ captured). In a techno-economic analysis, both capital costs (investment, installation, construction, and engineering) and operating costs (such as solvent makeup, maintenance, insurance, and taxes) could be considered. All process configurations could be compared to the conventional process regarding energy requirements, capital expenditures, and capture costs. Beyond the economic analysis, sensitivity analysis could also be conducted to gain a deeper comprehension of how economic variables impact the cost of CO_2_ capture.
